# Cost-Effective Synthesis of Bacterial Cellulose and Its Applications in the Food and Environmental Sectors

**DOI:** 10.3390/gels8090552

**Published:** 2022-08-30

**Authors:** Tahseen Kamal, Mazhar Ul-Islam, Atiya Fatima, Muhammad Wajid Ullah, Sehrish Manan

**Affiliations:** 1 Center of Excellence for Advanced Materials and Research, King Abdulaziz University, Jeddah 22230, Saudi Arabia; 2 Department of Chemical Engineering, College of Engineering, Dhofar University, Salalah 2509, Oman; 3 Biofuels Institute, School of the Environment and Safety Engineering, Jiangsu University, Zhenjiang 212013, China

**Keywords:** bacterial cellulose, cost-effective production, hydrogel, food sector, environmental applications

## Abstract

Bacterial cellulose (BC), also termed bio-cellulose, has been recognized as a biomaterial of vital importance, thanks to its impressive structural features, diverse synthesis routes, high thermomechanical properties, and its ability to combine with multiple additives to form composites for a wide range of applications in diversified areas. Its purity, nontoxicity, and better physico-mechanical features than plant cellulose (PC) make it a better choice for biological applications. However, a major issue with the use of BC instead of PC for various applications is its high production costs, mainly caused by the use of expensive components in the chemically defined media, such as Hestrin–Schramm (HS) medium. Furthermore, the low yield of BC-producing bacteria indirectly accounts for the high cost of BC-based products. Over the last couple of decades, extensive efforts have been devoted to the exploration of low-cost carbon sources for BC production, besides identifying efficient bacterial strains as well as developing engineered strains, developing advanced reactors, and optimizing the culturing conditions for the high yield and productivity of BC, with the aim to minimize its production cost. Considering the applications, BC has attracted attention in highly diversified areas, such as medical, pharmaceutics, textile, cosmetics, food, environmental, and industrial sectors. This review is focused on overviewing the cost-effective synthesis routes for BC production, along with its noteworthy applications in the food and environmental sectors. We have made a comprehensive review of recent papers regarding the cost-effective production and applications of BC in the food and environmental sectors. This review provides the basic knowledge and understanding for cost-effective and scaleup of BC production by discussing the techno-economic analysis of BC production, BC market, and commercialization of BC products. It explores BC applications as food additives as its functionalization to minimize different environmental hazards, such as air contaminants and water pollutants.

## 1. Introduction

Environmental pollution is an age-old phenomenon, and it remains a leading environmental cause of morbidity and mortality [1]. Waste materials generated by humans are at the forefront of global environmental pollution. Food waste particularly creates serious economic and environmental threats, globally. Approximately, one-third of all food produced worldwide goes to waste. In spite of economic losses, this food waste is one of the major contributors to greenhouse gas emissions. The wasted food discarded in landfills produces a large amount of methane, which is even a much more powerful greenhouse gas than carbon dioxide. The majority of these wasted food products can be effectively utilized or recycled. These wastes are extremely rich sources of important nutrients and sugars, which provide stupendous opportunities regarding their recycling and conversion into important products.

Cellulose is the most abundant natural polymer on Earth, obtained from plants [2], microorganisms [3], algae [4], and animals [5], and synthesized chemically [6] and enzymatically [7,8]. It is a homopolymer of disaccharides of anhydro-D-glucopyranose, known as cellobioses. These cellobioses are linked to each other through β-1,4-glycosidic linkages between the OH groups of C4 and C1 carbon atoms of the successive glucose moieties, and the individual units are present at 180 degrees, with respect to the neighboring moieties [9,10]. The molecular weight of cellulose is very high, and the value depends on its source and synthesis method and ranges from a few hundred to more than 20,000 Da [11,12]. The long cellulose chain is asymmetric and contains the reducing aldehyde (CHO) group, while the other end is non-reducing in nature [13]. Although cellulose obtained from different sources possesses the same chemical structure, its morphological, physico-chemical, thermal, and biological properties vary. Broadly, there are three classes of cellulose according to its source, properties, and synthesis methods, including cellulose nanocrystals, cellulose nanofibers, and bacterial cellulose (BC) or bacterial nanocellulose (BNC) [14]. Compared to the petroleum-based synthetic materials, the different cellulose types find applications in different fields, such as medicine [15,16], food [17,18,19,20], environment [21], textile [22], paper [23], additive manufacturing [24,25,26], pharmaceutics [27], cancer diagnosis [28], biosensing [29,30], and others.

Plants are the most abundant sources of cellulose, called plant cellulose (PC), which is present as lignocellulose, a tubular bundle of cellulose surrounded and entangled by hemicellulose and lignin, as well as containing some minerals [31], and it, thus, requires further processing, such as mechanical, chemical, and enzymatic treatment for the extraction of pure and high-quality cellulose [14]. On the other hand, BC is the purest form of cellulose, produced in vivo by a class of acetic acid bacteria [32,33] by utilizing different carbon sources and other medium components required for growth of bacteria. BC sheets are directly formed in the form of a hydrogel or membrane at the air–medium interface under aerobic conditions [34]. Cellulose synthesis in bacteria and plants is regulated by cellulose synthase (CS) operons, which code for the cellulose synthase (CS) complex. Besides the microbial cell systems, this purest form of cellulose is also produced in vitro by the cell-free enzyme systems by utilizing only the carbon source [7,8]. The cell-free enzyme system is a relatively simple approach for controlled and low-cost production of cellulose, due to the absence of bacteria and the available carbon source(s) can be transformed to cellulose. In all systems, i.e., plants, bacteria, and the cell-free enzyme systems, cellulose is produced in the form of β-1,4-glucan, which crystallizes and forms protofibrils, ribbons, and bundles [8,35]. All synthesis events are regulated by the CS complex [36]. The plants CS complex, unveiled in the freeze-fracture experiments, has a different organization compared to bacteria. The unique molecular configuration regulated by the bacterial cellulose synthase (*bcs*) operons gives different features to BC than PC, despite their similar chemical structures [37]. Furthermore, despite the same chemical structure of cellulose obtained from different sources, the micro- and macrofibrils of these celluloses differ in their morphological appearance and physiological behaviors [38]. Likewise, the unique synthesis pathway, extracellular transport across the terminal complexes (TCs), and in vitro self-organization of cellulose fibrils into high-ordered structures impart BC with distinctive morphological, physico-chemical, mechanical, thermal, and biological properties [39]. For instance, the reticulate three-dimensional (3D) fibrous structure, size and arrangement of fibrils, conformation, and other properties of BC differ from that of PC [39]. All such features impart BC with unique properties, such as high crystallinity, mechanical strength, water retention ability, aspect ratio, optical transparency, degree of polymerization (DP), and thermal stability, as well as moldability into a different shape, and make BC superior to PC for various applications, especially in the biomedical field, where cellulose of the highest purity is desired [34,40,41].

The applicability of BC has created a paradigm shift in the search for materials by exploring alternative cheap resources for its production instead of the current chemically defined glucose/fructose-based culture media [42]. Waste materials are excellent sources of various nutrients and organic compounds, and serve as commendable raw materials to be recycled into valuable bio-products, such as biofuels (biogas, bioethanol) and biopolymers (bio-cellulose, bio-plastic, and bio-films) [43,44]. The nutrients present in these waste products serve as the growth media for different microorganisms. Analogous to these facts, we utilized the waste of beer fermentation broth (WBFB) as the microbial source as the sole nutrient for bioethanol production in our previous study [45]. Furthermore, the sugar contents of WBFB could serve as the alternative source for BC production. Various cheap and commonly available carbon sources, along with waste materials, could be employed as prospective choices for the development of synthetic media.

In this review, we have summarized various low-cost and waste materials that could be exploited as alternative resources for the development of synthetic media for BC production. Previous individual reports have dealt with different alternative resources used for BC production, but herein we have summarized them, mainly focusing on cheap raw and waste sources for BC production. This review deals with the analysis of these resources in the context of their ability to produce BC. It is assumed that this report will be of worth not only for researchers but also for commercial BC producers.

## 2. Bacterial Cellulose History, Synthesis, and Structural Features

**Figure 1:**
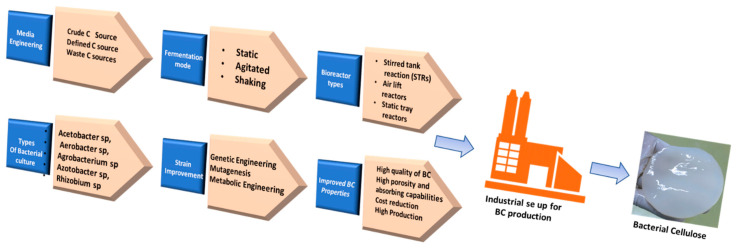
Illustration of various BC synthetic C sources, microbial strains, and synthetic strategies for high scale BC production.

The first report of BC production dates back to 1886, when A.G. Brown synthesized BC by using glucose as the main constituent; however, later, several other sources, such as galactose, fructose, and sucrose, were reported as the carbon sources for BC production [3]. Among the several bacterial genera, *Komagataeibacter xylinus* (formerly known as *Acetobacter xylinum*) and *Gluconacetobacter hansenii* are commonly used for BC production by utilizing glucose as the main carbon source [46,47]. BC production can be achieved through various synthesis routes by using a variety of bacteria from the genera *Aerobacter*, *Rhizobium*, *Achromobacter*, *Agrobacterium*, *Azotobacter*, *Acetobacter*, *Sarcina*, *Salmonella*, and *Escherichia*. Additionally, several wild-type strains have been modified for achieving a high yield and productivity of BC for scaling up the production processes (Figure 1) [43]. The BC production process can be broadly divided into the following two stages: the polymerization of glucose molecules into cellulose, followed by formation of the crystalline nanofibers through their self-assembling process. Bacterial species utilize the sugar present inside the culture medium to formulate glucose chains, which protrude out through TCs and form microfibrils through hydrogen bonding. TCs are aligned along the long axis of a bacterial cell. The cellulose molecules extrude from the TCs and assemble into a single elementary nanofiber of about 1.5 nm diameter. These elementary nanofibers then cluster into ribbon-like nanofibers of 3–4 nm thickness and with a 70–80 nm wide cross-sectional area. The ribbon-like microfibers form a web-shaped 3D reticulated network, forming a gelatinous pellicle floating at the air–medium interface of the culture medium [48]. New fibrils are reinforced from the media beneath the thick layer of BC. Agitation of the culture medium could also be employed for upgrading the BC production [49].

BC production in bacteria is regulated by *bcs* operons, comprised of four genes, named *cesA*, *cesB*, *cesC*, and *cesD*, which code for specific proteins. These proteins are not only involved in the intracellular synthesis of glucan chains but also regulate their extracellular transport across the external cell barrier (i.e., cell wall/membrane) and subsequent organization into high-ordered supramolecular structures [36]. BC can be produced in aerobic and anaerobic environments by the bacterial cells and the cell-free enzyme systems, respectively, which represent the in vivo and in vitro synthesis, respectively. In the biosynthetic pathway, the enzymatic reactions are mediated by specific enzymes, regulatory proteins, and various other cofactors produced within the bacterial cells. These intracellular reactions do not intervene with other anabolic activities, such as the synthesis of nucleic acid, lipids, proteins, etc., proceeding inside the cell. In both the cellular and cell-free enzyme systems, the anaerobic synthesis proceeds in the growth medium for the self-assembly of nanofibrils [44]. To maintain nanofibril production, the addition of specific cellulose-producing enzymes and cofactors is required to compensate for the absence of bacterial enzymatic reactions. Although the cell-free self-assembly of UDP-glucose has been studied extensively [44], the complex polymerization from medium to nanofibrils is yet to be fully unveiled. Subsequent sections thereby explore the challenges associated with the production of BC in cellular and cell-free environments.

The ribbon-like structure of BC is characterized by high purity and possesses a chemical resemblance to PC (i.e., C_6_H_10_O_5_)n [50]. It has high crystallinity and high-water holding capacity (over 100 times its dry weight). However, the actual crystallinity values of BC and PC can vary according to the analysis method, such as by determining the peak height, peak deconvolution, and amorphous subtraction determined through X-ray diffraction (XRD) or through C4 peak separation determined through NMR [51]. In the XRD technique, the crystallinity values may slightly deviate from the actual values, caused by the presence of small peaks besides the main crystalline peaks in the XRD pattern, which may raise the background noise and increase the overall amorphous and total area [52]. Furthermore, the crystallinity values of BC also depend upon the type of bacterial strain, synthesis method (static, shaking, agitation), and medium composition [35].

BC has different morphological and physiological behavior than PC, as it is devoid of lignin, hemicellulose, and pectin. BC production proceeds with two stages, where initially *A. xylinum* produces cellulose I, which is then transformed into thermodynamically stable cellulose II crystals through alkali treatment and urea as the additive [53]. During BC production by bacterial cells, the synthesized glucan chains align in the direction of cell movement and lock into the specific uniaxial crystalline arrangement to form a cellulose I structure. Cellulose I has two allomorphs, Iα and Iβ, which vary in their contents according to the cellulose source and differ from each other in their crystal packaging, molecular organization, and hydrogen bonding. In the absence of such control, i.e., bacterial cell movement, as in the case of cell-free enzyme systems [7,8], the randomly synthesized glucan chains in the culture medium fold into the thermodynamically stable cellulose II structure. The cellulose II polymorphic structure is also obtained via mercerization, through treatment of sodium hydroxide, or through regeneration [54,55,56]. The hydroxyl groups present on the cellulose surface are responsible for the hydrophilicity and biodegradability of BC, affording the possibilities of chemical modifications [57,58]. These hydroxyl groups offer hydrogen bonding sites for interacting with approximately 90% of water molecules, thus leading to a high water retention capacity.

According to the initial physical studies of BC structures, the BC pellicles were found to be stronger along the perpendicular direction of the fiber growth [59]. The first figures of Young’s modulus were recorded at 16–18 GPa across the surface of the BC plane, which was further improved up to 30 GPa by treating the sheets in alkaline and oxidative solutions [60,61]. It is increased in ribbon forms, which are oriented in a single plane upon pressing. Additionally, it increases with the increase in cross-sectional area, as observed in wider ribbons, where the effective cross-sectional area increases, creating an appropriate uniplanar orientation.

## 3. BC Production from Waste Resources

**Figure 2:**
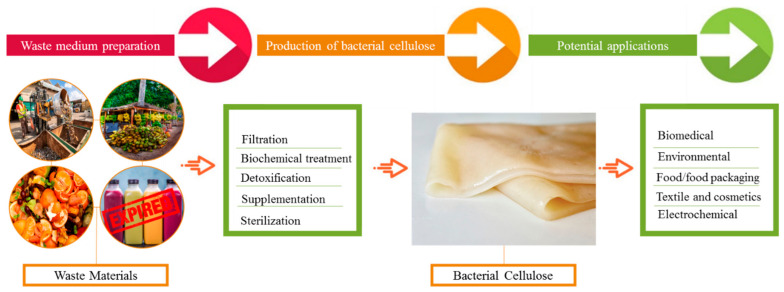
BC production from various waste sources and its potential applications in multiple fields.

Recently, numerous wastes have been reported for BC production; however, new resources are yet to be explored. Agricultural and industrial wastes are among the prime alternative carbon sources for BC production (Figure 2). Agricultural wastes, despite being a large source of global economic importance, are only used as a raw material for the production of value-added products in around 10% of cases [62]. Some important agricultural waste materials utilized in BC manufacturing are summarized in this article. Cornstalk hydrolysate is noted for its high sugar content, with 4.01 g/L of lignin, 1.84 g/L of mannose, 3.87 g/L of glucose, 2.95 g/L of furfural, 29.61 g/L of xylose, and 18.71 g/L of acetic acid. With the use of optimum pretreatment and detoxification conditions and the acetic acid pre-hydrolysate of cornstalk as the sugar source, Cheng et al. were able to produce 2.86 g/L BC [63]. The BC that was obtained had a fibril length of 300 nm and a diameter of 60 ± 10 nm. Another carbon source for BC production was rice barks [64]. Rice barks were first subjected to enzymatic hydrolysis and then utilized as a medium for BC production, yielding up to 2.42 g/L [64]. Another common agro-waste, wheat straw, is a valuable biomass that is commonly disposed of by burning it, resulting in significant air pollution. Wheat straw can be converted to a BC production medium using acid or enzymatic hydrolysis. For instance, [65] first performed the acid hydrolysis of the wheat straw prior to its utilization in BC synthesis. In another study, wheat straw was thermally and chemically treated to produce BC, which had up to 52.12 g/L carbohydrates.

Peels, which make up 5–40% of the weight of a vegetable or fruit, are inedible and are thrown away. Sugars, vitamins, and other vital nutrients are found to be in high amounts in these peels. Therefore, peels can be used as carbon sources for the economical manufacturing of a variety of value-added goods, including BC. Based on some previous studies, BC is also produced from the peels of various fruits and vegetables. The waste of peels from the orange juice industry has been used as a medium for BC synthesis. About 8–10% cellulose, 10% moisture, 5–7% hemicellulose, 30–40% sugar, and 15–25% pectin make up the orange peels. Enzymatic hydrolysis of orange peels using cellulose and pectinase boosted the sugar content to 60–80 g/L, resulting in 4.2–6.32 g/L BC, a substantially higher concentration [66]. In our recent study, 3.71 g/L BC was produced by G. hansenii after 7 days of cultivation by utilizing waste tomatoes as the low-cost substrate [67]. In another study, the textile waste old nylon-6,6 was biotransformed into BC by the *Taonella mepensis* WT-6 strain [68]. The utilization of a type of waste named jaggery (gur) from sugarcane was effectively converted to BC, with good morphological and physiological properties [69]. Oat hulls are also a low-cost and renewable source of carbon. These make up 28 percent of the grain’s weight and contain 45 percent cellulose [70]. Large amounts of oat hulls are produced by the oat processing businesses, which produce oat cereals, cookies, and slices. Even when grown in different climates, oat hulls have the same mechanical properties and chemical composition (same strength, elasticity, and size), making them globally uniform and an industrially viable waste. The oat hulls can, thus, be used to establish a standardized media for BC synthesis. Because the BC-producing bacteria cannot use the oat hulls directly as a carbon source, other pretreatment processes are used to hydrolyze them into fermentable sugars. A four-step technique for producing BC from oat husks has recently been disclosed.

The wastewater of various fermentation industries, including butanol, acetone, and ethanol, etc., is a rich source of organic acids and sugars, which can be effectively utilized in BC production media development. One study reported 1.34 g/L BC production by using fermentation wastewater. [71]. Furthermore, wastewater from the beer fermentation industry, lipid fermentation, food industry, and backing sectors is also highly rich in sugars and proteins. Waste of beer fermentation broth was utilized as fermentation media for BC production and up to 7.02 g/L BC was produced. [45]. Another group of researchers applied the wastewater from lipid fermentation as the sole carbon source for BC production and they produced 0.659 g/L BC on the 5th day of incubation [72]. Besides these wastes, recently, Ul-Islam et al. reported a comparative production and physiological analysis of the BC production from waste juices, coconut water, and sugarcane juices. The BC produced from various waste sources exhibited comparative morphological and physico-chemical features to the control BC produced from refined media [39]. A shift towards applying alternative carbon sources will not only reduce the BC production costs, but also will play an important role in resolving the environmental issues associated with waste.

## 4. Techno-Economic Aspects of BC Production and Developing BC-Based Products

The techno-economic analysis of BC production and its utilization in developing various functional materials can be discussed from aspects of commercialization of BC, the BC market, and feasibility of large-scale production, as discussed below.

### 4.1. Commercialization of BC

Currently, the commercialization of BC and BC-based products has been limited by the following two main factors: the limited innate structural and functional properties of BC to meet the desired material properties for specific applications and the high BC production cost. As BC mainly finds applications in the biomedical field, the commercialization of BC and BC-based products could be discussed from this aspect. Pristine BC does not possess antimicrobial, antioxidant, anticancer, or electromagnetic properties [73,74]. Moreover, although it is non-toxic and is similar to extracellular matrices, pure BC has limited biocompatibility because it lacks adhesion sites to support the adhesion of cells. Due to these limitations, pure BC cannot be used for the development of wound dressings, drug delivery systems, synthetic organs, grafts, implants, etc., and requires functionalization with other materials, such as polymers, nanomaterials, clays, antibiotics, peptides, etc. [48,75]. Likewise, due to lacking electromagnetic properties and limited optical transparency, pure BC cannot be directly used in the development of magnetic and optical devices [76,77]. Similarly, the high production cost of BC due to expensive medium components is another major limitation in the commercialization of BC-based products. In addition to the high cost of medium components, the low yield and productivity are other factors that limit the scale-up of the process. The issue of high production cost due to the use of expensive medium components has been addressed, to some extent, by exploring the potential of various agro-industrial wastes and low-cost substrates [78]. On the other hand, the yield and productivity of BC have been increased to considerable limits by designing advanced reactors and exploring efficient bacterial strains, as well as engineering novel strains [42]. It is assumed that by overcoming the production cost and enhancing the yield and productivity, BC-based products can gain a place in the market in the near future.

### 4.2. BC Market and Selling Price

Several BC and BC-based products are already on the market. Among others, nata de coco is the main commercial form of BC that is used as the main food component in several countries. Its use in food was started in the 1980s by Del Monte Corporation by adding nata de coco in a tropical mix dessert. Presently, Japan, United Arab Emirates, China, Canada, Malaysia, and the Philippines are the major players in the BC market. According to an estimate, the BC market is expected to increase from USD 207.36 million in 2016 to USD 497.76 million by end of 2022 and further reach USD 700 million in 2026, with a capital annual growth rate of 15.71% [79]. Celluforce, Innventia AB, American Process, University of Maine, Borregaard, US Forest Service, and Nippon are among the key players in the BC market spread to different countries, such as the USA, China, Canada, European Union, and Japan.

Nada de coco is commercially available in the form of slabs or diced pieces, as well as the remains obtained during the trimming process, called ‘reject’ nata de coco. The current price of nata de coco ranges between USD 200 and 1000/ton, which equals to USD 1–10/kg on a small scale [79]. However, this pricing varies among the manufacturers and is dependent on the final finishing of the product. For example, the price of slabs and slices varies between USD 0.31–0.36/kg and USD 0.27–0.36/kg, respectively, while ‘reject’ nata de coco is sold at a retail price of USD 0.33–0.45/kg. It is expected that the utilization of different wastes and low-cost substrates as the production media would greatly reduce the selling price of BC and would provide a boost to the BC market.

### 4.3. Techno-Economic Analysis for Large-Scale BC Production

The large-scale production of BC is often hindered due to a lack of information on process feasibility. Several studies have carried out techno-economic feasibility analyses of BC production. The BC production process at an industrial scale could be divided into the following two steps: upstream processing and downstream processing. The upstream process involves the preparation of pre-culture called the ‘seed fermentation’ and the static fermentation, referring to BC production. The downstream process involves the washing, drying, and modification of BC into the desired size and shapes (e.g., filaments, cubes, slabs, etc.). Technical analysis of industrial-scale production of BC involves a literature survey, material balance, and resources estimation, and requirement optimization, while the economic analysis involves the estimation of capital and operational cost (investment, operation cost, maintenance, taxes) and cash flow analysis (sale, taxes, gross and net profit).

A recent study [80] carried out a techno-economic feasibility analysis of a 60-ton annual capacity facility of Kombucha-based BC production during the fermentation process by using the Super-Pro Designer. The findings showed that this facility requires USD 13.72 million and USD 3.8 million as the estimated investment and operating costs, respectively. Out of this cost, 89% covers the facility and labor costs. The output results show that this facility has a payback time of 4.23 years, and 23.64% and 16.48% return on investment and internal rate of return, respectively. Using the same Super-Pro Designer (v9), Duorado et al. [81] carried out a techno-economic feasibility analysis of large-scale production of BC by utilizing waste molasses and a chemically defined medium. They used the data from a study by Keshk et al. [82] for the production of BC over a 7-day fermentation period. The facility can accommodate 60,000 L/month of culture medium and produce 42 tons/month or 504 tons/year BC. The facility cost was estimated to be USD 13 million, of which 71% covers the equipment and installation cost and 29% covers the engineering and construction cost. An additional USD 0.966 million was reserved for contingency charges, such as those for estimation error, price fluctuation, minor process changes, unpredicted cost, etc. The annual production cost was estimated to be USD 7.4 million, of which USD 2.1 million covers the direct operational cost, USD 2.18 million covers the labor cost, USD 2.1 million covers the fixed charges (taxes, insurance, rent, etc.), and USD 1 million covers the overhead cost (food, medical, administration, accounting, etc.). The study estimated an annual profit of USD 3.3 million, with a payback time of 4 years. These values are flexible and the input cost can be minimized by increasing the capacity of the operation unit and utilizing agro-industrial wastes and low-cost substrates. Furthermore, automating the process would result in reducing the energy and labor cost. These techno-economic analyses show that the industrial-scale production of BC is highly capital-intensive.

## 5. BC Applications

Due to the unique structural properties exhibited by cellulose of bacterial origin, its applications have been focused on in different fields. At first, White and Brown evaluated BC for its commercial applications [83]. Unlike PC, no harsh chemical processing is needed for BC. Its high-water retention capacity allows the formation of never-dried BC membranes, and hence proved to be a potential hydrogel. A wide range of morphological properties could be obtained depending on the synthesis method, as well as the substrate. Several applications as materials have been reported in the industry, such as headphones, paper, and textiles. Their significant Young’s modulus, as estimated by their excellent water maintenance capacity, accompanied by high internal loss, identifies them as an ideal material for speakers’ diaphragm. Sony Corporation has marketed high-fidelity loudspeakers and headsets, with the use of BC as an acoustic diaphragm [84]. The use of BC resulted in high sound velocity, comparable with that of aluminum and tin, as well as low dynamic loss [85]. The addition of BC pellets into paper resulted in a four times increase in its tensile strength, in comparison to regular paper [86]. Cheng et al. reported a high Young’s modulus and mechanical strength of CMC-BC paper, in contrast to pulp paper [87]. Fernandes et al. reported saturation of BC membranes with Baygard EFN (perfluorocarbon) and Persoftal MS (polydimethylsiloxane), known as hydrophobic polymers utilized in the textile industry for finishing processes. The vapor permeability values, drop absorption over time, water static contact angles, and improved mechanical strength of the hydrophobized, malleable, robust, and breathable nanocomposites based on BC thus obtained featured all the favorable properties to be applicable in textile industries [88]. Its biocompatible and biodegradable properties have led to a greater trend in the pharmaceutical, medical care, and cosmetic industries [89,90,91,92,93]. Weyell et al. studied doxycycline hyclate@BC as a wound dressing in dentistry and its application in controlled drug delivery [94]. Due to the biocompatibility of BC with skin tissues, permeability, and high water retention properties, they have been used in the preparation of moisturizing and anti-aging skin masks [95]. Chen et al. studied nitrated BC, which served as a homogeneous 3D porous structure for the incorporation of explosive particles. The cross-linked gel matrix was found to restrict crystal growth, and hence resulted in the formation of nanocomposites [96]. In the past, due to the high cost of BC produced by traditional Hestrin and Schramm (HS) medium, as compared to food products’ own value, their applications in the food industry were restricted. With the production of BC from cost-effective fruit fermentation media, utilization of BC in food products has significantly increased. Accepted by the USA Food and Drug Administration in 1992, BC is a type of dietary fiber, and is classified as “generally recognized as safe” (GRAS). The health benefits of dietary fiber are well-known and can reduce the risk of chronic diseases, such as obesity, diabetes, cardiovascular disease, and diverticulitis. In this review, applications of BC in the food industry and environmental sector have been highlighted in detail.

### 5.1. BC Applications in the Food Industry

#### 5.1.1. Raw Food Material

BC is well-known for its application as a raw material in nata de coco, a traditional dessert of Asia. BC sheets procured from coconut milk, or coconut water, are drawn into cubes, followed by their immersion into sugar syrup [97]. In a similar manner, wide-ranged colors and flavors of nata products can be developed, using BC sheets obtained from different valued fruit juices. Nata can be obtained in variant shapes by making use of different vessels [98]. Additional processing techniques greatly vary the texture of the product. For instance, freeze-drying results in crunchy nata de coco, while conventional oven drying gives a foam-like texture to the nata. For example, nata de pina and nata de soya are produced from pineapples and tofu whey, respectively [97,99]. In addition to their direct consumption as a dessert, nata de coco is also added to jellies, yogurt, and beverages for texture enrichment [100]. Moreover, BC has been found in Manchurian tea or Chinese kombucha. Kombucha tea is obtained as a result of sugared tea fermentation in a microbial culture with symbiotic relation between lactic acid bacteria, yeasts, and *Komagataeibacter* bacteria. Due to the slightly acidic and carbonated taste of the drink, it has resulted in high customer demand. Contamination of the beverage is prevented by certain antibacterial agents produced as a metabolic by-product in the symbiotic culture. It is also considered as a high fiber beverage and the low-calorie active agent reduces lipid accumulation and hereby protects the liver against damage. It has been reported that it possesses therapeutic and immunomodulatory properties. Casaburi et al. reported BC as a raw material for the synthesis of carboxymethyl cellulose (CMC). CMC is used as a thickening, stabilizing, and binding agent in different food and beverages [101].

#### 5.1.2. Food Ingredient

**Figure 3:**
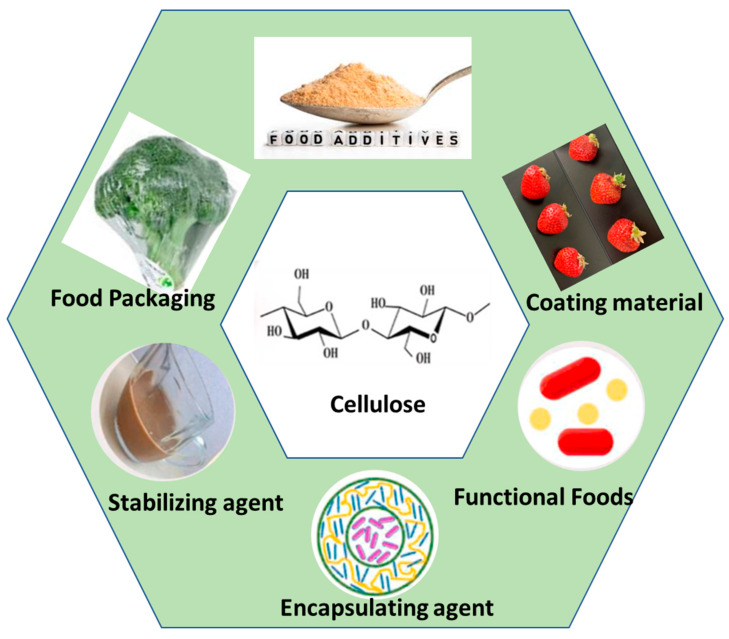
Bacterial cellulose applications in the food industry and as food packaging materials. Figure reproduced from [108], with permission from the publisher.

Due to the extraordinary structure and properties of BC, it has found applications as an active ingredient in food formulations. BC is capable of modifying rheological properties when used as a food ingredient. This behavior is found to be due to the superior application of BC as a gelling, thickening and stabilizing, and water-binding agent [102]. BC has been reported as an economic alternative to the commonly used thickening agent for WPI emulsion [103]. Okiyama et al. reported that BC can solidify crumbly food hydrogels, and thus, in this way, serves as a potential filling material. When added to pasty food materials, BC significantly enhanced the texture by decreasing the extent of stickiness, therefore allowing quantitative spooning of the food item [104]. Following its addition to chocolate drinks, the BC matrix holds cocoa particles suspended throughout their structure, and hence prevents their deposition at the bottom. BC is capable of significantly improving the firmness and texture of tofu, by increasing its gel strength with a mere addition of 0.2–0.3%. Moreover, the addition of BC to kombaboa enriches its tensile strength and also attempts to improve its fragile and ductile nature. Thus, BC-modified kombaboa can go through the aging process, while maintaining its sensory evaluation properties [105]. BC has potential functions as an ice cream ingredient, where it tends to modify its texture by supporting its structure, enhancing the viscosity, retention time, and hence its meltdown time [106,107]. Thus, it is clear from the above studies that BC is able to stabilize processed foods over a wide range of pHs, temperatures, and snowpack environments. Along with stability, BC also functions to improve the quality and storage conditions of canned food. A few BC applications in the food industry are illustrated in Figure 3.

#### 5.1.3. Food Stabilizer

Pickering emulsion refers to a phenomenon where solid colloidal particles are able to be adsorbed at the interface, separating two non-miscible liquids, thus forming a strong monolayer at the liquid–liquid junction [109]. This inter-liquid layer tends to avoid their mixing, hence stabilizing the emulsion drops. In comparison to the conventional emulsion, Pickering emulsions have several advantageous characteristics, such as reduced toxicity and foaming problems, as well as lower environmental impacts. Unlike classical emulsions, where the addition of surfactants is required, they are able to stabilize the emulsion in the absence of surfactants. In both cases, stabilization is caused by the anchoring of solid particles on the droplet surface. Even so, the accumulation of solid particles on the interface follows a different mechanism, wherein the particle surface is partially dampened by water and oil. Therefore, solid particles are typically needed to be amphiphilic, but not necessarily [110,111,112].

BC and BCNCs with excellent amphiphilic properties have been studied as a stabilizer in surfactant-free Pickering emulsions. The presence of a large number of hydroxyl groups results in their hydrophilic character, while the hydrophobic nature is associated with the extensive hydrogen bonding and crystalline organization of the polymer chains [113]. Paximada et al. studied BC as a stabilizer for oil-in-water O/W emulsions (with 10% olive oil). Better stabilizing capacity was observed for BC in comparison to HPMC and CMC, due to the extraordinary capability of BC to form a strong visco-elastic network between the oil droplets [114]. In another study, they studied an oil-in-water emulsion containing 5% olive oil, where whey protein isolate (WPI, 2–5 wt%) was utilized as a surfactant and BC (0–1 wt%) was added as a stabilizer. At a lower concentration of BC, about 0.5 to 0.7%, the emulsion was unstable, while with the addition of 1wt% BC, the emulsion stability increased considerably, thereby preventing coalescence [103]. Zhai et al. studied an O/W Pickering emulsion by using peanut oil with a percentage concentration of 15% by volume, and 0.05% *v*/*w* BC nanofibres were added as the stabilizing agent. Nanofibers derived from BC significantly reduced the surface tension of oil/water droplets from 48.55 ± 0.03 to 34.52 ± 0.05 mN/m. These food-grade Pickering emulsions have great potential to deliver lipophilic bioactive substances in the food industry [110]. Tempo-oxidized BC (TOBC) has been used as a food stabilizer in surfactant-free Pickering emulsions. Due to its smaller size as compared to BC, TOBC was more effective in stabilizing the oil/water interface. With a fibril dosage of 0.7%, the emulsion did not experience coalescence or creaming over eight months. The greater electronic repulsion and zeta potential values were manipulated by fiber size and its wettability, thus rendering greater stability to the emulsions. Due to the smaller particle size and higher zeta potential values (derived from sulfate groups), BCNCs synthesized by hydrolysis of BC by sulfuric acid showed better stabilizing ability for O/W emulsions, as compared to BCNFs [115]. Kalashnikova et al. investigated BC nanocrystals (BCNCs) as a stabilizer in hexadecane/water emulsions. The mono-dispersed oil-in-water droplets with a diameter of 4µm were stable for up to several months. The droplets with a percent coverage above 60% showed greater stability, due to the irreversible adsorption of particles in the formation of a 2D network [116]. A dry BC/CMC formulation with a zeta Potential value of (−67.0 ± 3.9) mV and a volume median diameter of (601 ± 9.7) μm was employed as a stabilizer in an isohexadecane-in-water emulsion. The synthesized material was capable of decreasing the oil/water interface energy [117].

#### 5.1.4. Fat Replacer

In terms of providing foods with rheological, physical, and texturing properties, fats are recognized as an important ingredient in a wide variety of food products. For instance, in cakes, fats keep air cells fixed tightly in the oil-in-water emulsion, thus offering them softness, volume, and flavor [118]. On the other side of the coin, a number of health-related problems, such as obesity, heart diseases, diabetes, and high blood cholesterol levels, are found to be associated with high-fat doses [119]. Thus, numerous endeavors have been coordinated to lessen the fat substance of food items. As a consequence of progressive awareness amongst customers with regard to the destructive impacts of foodstuff with high-fat content, customers have requested low-fat products [120]. However, there is a widely held view that low-fat food varieties are less alluring in comparison with their high-fat analogs, which has been attributed to the poor sensory effects of the reduced-fat diet [121]. Thus, researchers are searching for suitable fat replacers, which can satisfy the demand for both the structural and sensory properties. Fat replacements reduce the food calories, mimic the fat functions and are also helpful in avoiding health issues thought to be related to diets rich in fats. Different hydrocolloids have been studied so far in a number of food products, such as cakes, cheese, meat, etc. [122,123,124]. Due to the unique structural properties of BC, such as its high purity, and crystallinity, greater tensile strength, degree of polymerization enhanced water-retention capacity, and strong biological adaptability, they have found applications as a fat replacer in a variety of dietary products [105,125]. For example, Guo et al. reported nano-BC/soy protein isolate (SPI) as a potential cream substitute in ice cream. The nano-BC/SPI effect on rheology, texture, and sensory characteristics of ice cream was studied [107]. Another study reports the utilization of BC in *Turkish Beyaz*, a fat-reduced cheese. An increase in melting time, softness, and sensory properties was observed for BC-based low-fat cheese in comparison with the control counterpart [126]. Akoğlu et al. studied the effect of BC on the protein and moisture content of Turkish sausage, named succuk. With the increasing levels of BC addition, % protein and moisture content increased, accordingly. With higher BC amounts and reduced-fat contents, the hardness of the sausage reduced without any significant change in texture, color, odor, or flavor [127]. In another study, Akoğlu et al. investigated BC for its function as a fat substitute in mayonnaise. They reported acceptable results for low-fat mayonnaise, in terms of rheological properties and sensory features [128].

#### 5.1.5. Meat Analog

BC is used for the synthesis of vegetarian meat, which is well-known as an analog for natural meat [129,130]. BC, in alliance with Monascus extract (which is obtained from a naturally occurring pigmented mould, *Monascus purpureus*), has been used for the preparation of non-animal meat. *Monascus purpureus* yields colored polyketide pigments that are red, orange, or yellow [131]. It is also known to secrete antihypercholesterolemic agents, including mevinolin and monacolin [132]. Colored BC products have been obtained by the fermentation of BC with that template. These tinted composites were then used as a raw material for various food products, principally for meat analogs [133]. The composite is highly resistant to color and morphological changes; also, its flavor resembles that of animal meat. Vegetarian meat prepared from the BC/Monascus complex has found appeal in the market for a number of different reasons, as Monascus offers a cholesterol-lowering effect, BC is a dietary fiber, and has a non-animal origin. The product as a potential substitute for meat products is highly attractive to diet-conscious individuals [134].

#### 5.1.6. Food Packaging

Food packaging is considered fundamental in food preservation and in ensuring minimal food spoilage and wastage. These packages function as potential barriers against the action of contaminating agents, physical damages, and other environmental factors on the quality and safety of food products [135]. Traditionally, plastic-based packaging materials, such as polyethylene terephthalate, polyethylene, polypropylene, polystyrene, and polyvinyl chloride, are used. Although they are capable of exhibiting superior packaging properties, their non-biodegradable nature and limited recyclability application have made them a serious threat to the environment. In order to meet the customers’ demand for alternative biocompatible packaging material and to reduce environmental crises caused by non-sustainable polymers, biodegradable, natural polymers have been introduced. These natural polymers control the exchange of gases, unpleasant aromas, humidity, and the immigration of unwanted compounds. Furthermore, they are known to release certain types of antioxidant and antimicrobial agents [136]. This provides an additional stress factor for food preservation [137]. Amongst many others, BC has found various applications in food packaging due to its differentiated properties from the other known polysaccharide-based polymers [136,138,139,140]. Edible sheets and films obtained from BC nanofibrils can be made into food wrappers and packaging films. Viana et al. synthesized BC nanofibril and pectin-based edible films in varying proportions. They reported greater strength and water resistance properties for films with higher BCNF concentrations [141]. BC films have high mechanical strength and flexibility, but there is a lack of antimicrobial activity [142,143]. In order to impart antimicrobial activities, various approaches have been put forward. Abral et al. reported the synthesis of starch/chitosan-based BCNF films, which displayed remarkable water moisture resistance, barrier properties, and antibacterial activities. The characterization results suggested that the prepared films were highly effective regarding their application in food packaging [144]. According to some studies, the presence of food-grade antimicrobial agents contained in the BC films, such as nisin and lactoferrin, is held responsible for their antimicrobial action [145,146]. Nguyen et al. (2008) impregnated BC films with nisin solution and studied their ability to control the growth of total aerobic bacteria and Listeria monocytogenes on vacuum-packaged frankfurters. Their efficiency was evaluated in relevance to nisin concentration and total impregnation time [147]. Antimicrobial agents used directly in active packaging are very expensive. Rollini et al. synthesized bacteriocin sakanin-A/BC nanocrystals and studied their application as antibacterial food packaging. The bacteriocin was prepared from cheese whey protein (CWP), a cheap substrate obtained as a by-product, thus proposing an economic approach for active packaging [148]. Castro et al. loaded curcumin and carvacrol onto cyclodextrin (CD)-grafted TEMPO-oxidized cellulose nanocrystals (TOCNCs). The results demonstrated prolonged antimicrobial activity for TOCNC-COOH/HPβCD in comparison to pristine TOCNC-COOH [149]. Panaitescu et al. investigated a PHB–BC–ZnO film, produced by plasma treatment, as a potential candidate for green and sustainable packing material [150]. Tsai et al. synthesized SYM-zein/BC nanocomposite films by adsorption of silymarin (SYM) and zein nanoparticles over the surface of BC nanofibers. Their potential application for food packaging was revealed by the extended shelf life of the preserved fish [151]. A BC and silver nanoparticle (AgNPs) composite was demonstrated to be an antimicrobial material for food packages by Yang et al. [152]. Taking into consideration the above-discussed investigations, BC has been uncovered as a fascinating and promising biopolymer for the advancement of materials with regard to their applications in the food packaging industries.

### 5.2. BC Applications in Environmental Sectors

#### 5.2.1. Pollutant Absorption/Absorption of Organic Solvents

**Figure 4:**
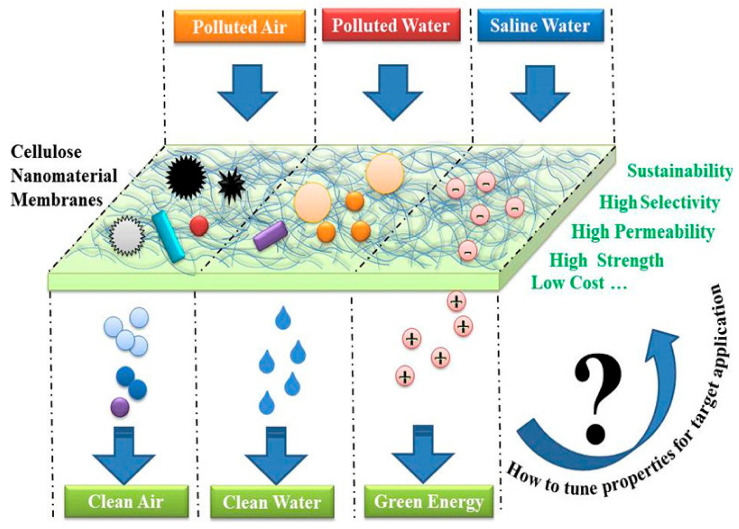
Schematic illustration of using cellulose nanomaterials for achieving clean air, water, and green energy. Figure reproduced from [157] with permission from publisher.

Organic solvents associated with a number of environmental, economic, and health issues have become a major concern in the last few decades. The separation of hydrogen and carbon-based solvents has become a particular interest of environmentalists. The high purity, crystallinity, and high aspect ratio of BC mean that it has been classified as an interesting material for the absorption of organic solvents. Ultrafine BC/graphene oxide (GO) aerogels were prepared by a freeze-drying technique, which, after further treatment with H_2_, were converted to BC/rGO. The nanocomposite aerogels exhibited a specific absorption capacity for organic solvents only [153]. In situ synthesis of carbon nanofiber biosorpent from BC and its simultaneous magnetization have been reported. The BC-based magnetized CNFs with a mesoporous-macroporous structure, large surface-to-volume ratio, and suitable adsorbent/adsorbate association showed excellent efficiency for the removal of bisphenol A [154]. Khamkeaw et al. reported the preparation of mesoporous zeolite by utilizing BC-AC500 as a template and studied its application for successful adsorption of formaldehydes [155]. Pereira et al. investigated the functionalization of BC aerogels by the TEMPO oxidation method, followed by salinization with trimethyl silane. These aerogels were reported to show selective absorption capability for organic liquids [156]. Various applications of BC and BC-based composites in environmental sectors are shown in Figure 4.

#### 5.2.2. Filter Membrane for Water and Air Purification

Air pollution caused by particulate matter, biological materials, and chemicals has become a global concern. These pollutants are recognized as a basis for a number of diseases in living organisms, mainly human beings. PM is composed of variable-sized particles and liquid droplets. Generally, PM with a diameter smaller than 2.5 is classified as PM_2.5_, and that with a diameter in the range of 10 µm is termed as PM_10_. PM_2.5_ with an extremely small particle size is easily penetrable into the bronchi and lungs; thus, it is recognized as the most hazardous pollutant. Long-term exposure to PM_2.5_ results in the accumulation of plaques inside the arteries; thus, it may cause arteriosclerosis and vascular inflammations, which would eventually lead to cardiovascular diseases, such as heart attacks. As people spend most of their time inside buildings, in order to improve indoor air quality, air filtration systems have been installed inside buildings. For the maintenance of active air exchange, highly efficient eco-friendly air filters, which typically show little air resistance, are required. Air filters are also used for various purposes, including residential purposes, general commercial purposes, personal masks, hospitals, automotive industries, etc., for the eradication of air pollutants. Petroleum-based or chemically synthesized polymers are conventionally used in air filters, for example, polypropylene (PP), polyethylene (PE), glass fibers, etc., but the disposal of these traditional air filters consequently led to the production of secondary pollutants. Therefore, scientists are highly interested in the utilization of biomaterials as air filters. Biocompatible, cost-effective and highly pure BC with a uniform structure, and extraordinary physical and mechanical properties has been utilized in air filters. The 3D-nano network of BC allows physical filtration of PM. Two potential biomaterials, namely soy protein isolate and BC, have been employed as highly efficient air filters. The 3D nanostructure of BC is held responsible for the physical trapping of PM from the air, whereas the functional moieties on SPI further capture the PM by dipole interaction and electrostatic attraction [158]. Wu et al. synthesized silver nanowire (AgNW)-BC air filters with antibacterial activity, increased porosity, and air permeability. The in situ cultivation method was employed for the incorporation of AgNW into the BC matrix. The high filtration efficiency of 99.7 and 99.8% for PM_2.5_ and PM_10_, respectively, was attributed to the electrostatic adsorption achieved by AGNW incorporation [159]. He et al. reported an even distribution of modified soy protein isolate (SPI) on BC membranes. MSPI-BC composites exhibited a highly stable structure and air filtration efficiency, thus presenting an environmentally benign and potential air filter [160].

Oily water is produced as a consequence of emulsified effluviums from various industries, such as chemical, petrochemical, mineral activities, and oil spills. Oil-in-water emulsions and oily water are among the main environmental pollutions, whose treatment has become a global concern. By definition, emulsions are complex mixtures of oil, water, and other additives, including emulsifiers, anti-foaming, and corrosion inhibiting agents. Unlike free-floating oils, which can be easily separated by effortless physical processes, emulsions are chemically stable systems and require suitable methods for their removal. For this purpose, membrane filtration, such as microfiltration, nano-filtration, and ultra-filtrationm has been introduced in the literature. In most cases, commercially available membranes are synthesized from synthetic polymers, which in turn require a large number of organic solvents in order to be prepared. Hence, biopolymer-based membranes have found popularity among researchers. BC is a strong, biodegradable biocompatible, and renewable polymer. The reticulated structure of BC with a high density of small pores means that it is a promising material for filtration membranes. Three-dimensional web-like BC aerogels (BCAs) have been prepared by surface modification of BCNFs with trimethylchlorosilane, followed by freeze-drying. The hydrophobic BCAs thus produced have been investigated as selective filter membranes for oil absorption [161]. Galdino, Jr. et al. reported the synthesis of BC pellicles from *Gluconacetobacter hansenii* in an alternative medium. The BC membranes revealed high thermal stability, flexibility, and mechanical strength, thus showing 100% oil removal efficiency from oil-in-water emulsions [162]. In a similar manner, Hassan et al. studied the removal of oil from oil-in-water emulsions by never-dried BC and cross-linked BCNFs [163]. A superhydrophobic/underwater superoleophobic filtration membrane has been designed for the removal of oil. BCNFs were blended with silica microparticles, followed by coating with polydopamine (PDA) [164]. The BC/SiO_2_-MPs/PDA O/W separation membranes exhibited >99.9% removal efficiency, with a flux rate of ~10,660 LM^−2^h^−1^. Hu et al. prepared antifouling ultrafiltration BC-based membranes by modification of a nanofiber network with mussel-inspired dopamine, followed by coalescence with two-dimensional graphene oxides [165]. Similarly, Alves et al. prepared BC membranes and investigated their application in industrial and environmental wastewater remediation [166].

### 5.3. Bio-Adsorbent for Heavy Metals

Due to the notorious toxicity and daunting biodegradation of heavy metal ions, heavy metal pollution has been raised as a global concern amongst environmentalists. These metal ions may enter into food chains and reach living bodies, where they readily tend to accumulate and might lead to serious threats to living beings. Among the different well-known techniques for the removal of these toxic ions, adsorption has attracted greater attention, bearing the advantage of the ease of operation, without the production of secondary pollutants or any by-product. BC with high porosity, remarkable water retention capacity, highly crystalline nature, and mechanical strength has been suggested as an efficient bio-adsorbent for metal ion removal. So far, BC has been utilized as an adsorbent for various metals, such as cadmium, lead, mercury, copper, etc. [167,168,169,170,171]. BC and poly(amidoxime) aerogels (BC/PAO) have been investigated for the efficient removal of heavy metals, taking into account the presence of a high number of amidoxime functional moieties and the interconnected porous three-dimensional structure of BC/PAO aerogel [172]. Hosseini et al. studied a BC/PANI aerogel as a bioadsorbent for the detoxification and adsorption of hexavalent chromium ions. Along with adsorption, it also reduced the Cr(VI) into its less toxic Cr(III) form [173].

### 5.4. Protein Adsorption

Protein adsorption behavior has been recently paid more and more attention. As a matter of fact, such behavior is governed by the chemical structures of material surfaces. Therefore, surface modification may provide the appropriate behaviors of the protein–surface interactions needed. The large surface area of BC, due to its fine network structure, makes it a suitable bio-adsorbent for bio-macromolecules, such as protein. Various literature studies show that BC demonstrates a greater affinity for the adsorption of cellobiose dehydrogenase, as compared to cellulose derived from wood pulp [174]. In another study, phosphorylated bacterial cellulose (PBC) and phosphorylated plant cellulose (PPC) were utilized for the adsorption of amines and proteins [175]. PBC exhibited a greater adsorption capacity than PPC. Nilde et al. studied quaternary ammonium BC and PC as bio-adsorbents for hemoglobin at a basic pH. It was reported that the protein was adsorbed more efficiently on QABC through electrostatic interactions [176]. Oshima et al. reported adsorption of lysosomes on phosphorylated BC. The protein molecules were adsorbed on the PBC by electrostatic interaction at a lower pH, relative to their iso-electric points [177]. Lin et al. modified BC membranes by carboxymethylation reactions with varying DS and utilized them for the adsorption of proteins, taking bovine serum albumin (BSA) as a model. The chemical structure of CBC and its electrostatic interaction with BSA proved it as an efficient adsorption material. Furthermore, CBC membranes with high DS were reported to have greater adsorption capacity [178].

### 5.5. Catalytic Support for Pollutant Degradation

Toxic organic compounds, including dyes and nitroarenes, are water soluble and are a serious threat to water bodies, and hence lives. Different strategies have been employed for the degradation of these potential pollutants. Amongst the various strategies employed for the degradation of these potential pollutants, nanocomposite catalysts have been widely applied. Despite their significant catalytic activity attributed to the high surface-to-volume ratio of nanoparticles, their recovery from the reaction medium is challenging. In addition, these nanoparticles are at a higher risk of agglomeration, which consequently reduces their catalytic performance. BC with a high surface area, provided by the nanofibers within the size range of 50 to 100 µm and high density of –OH functional moieties, emphasize its use as a catalyst supporting material. Furthermore, they increase the surface area, due to its porous and nanofibrous structure; thus, a greater number of active sites are available for reaction molecules to adhere to and increase the catalytic activity. The water-retention capacity and stability of BC in an aqueous medium have been attributed to inter-molecular and intra-molecular hydrogen bonding, thus ensuring their ease of handling, recovery, and recyclability, the characteristics of a promising catalyst. Its highly porous structure and insolubility in water allow a large number of reactant molecules to enter into the 3D matrix and interact with the embedded catalyst. BC has been reported as a catalyst support in a number of pollutant degradation processes, such as chemical reduction and photocatalysis, Fenton reactions, ozonolysis, etc. Wibowo et al. investigated the synthesis of a BC@Fenton catalyst, where a BC hydrogel was dipped into Fe^2+^ and Fe^3+^ (1:2) precursor solutions, followed by immersion into ammonia solution overnight. The particle size of the heterogeneous catalyst was greatly reduced by BC, and as a consequence, the catalytic efficiency of the BC@Fenton catalyst in the degradation of methylene blue was enhanced [179]. Song et al. investigated BC as a supporting matrix for the synthesis of copper and nickel nanoparticles and evaluated their catalytic efficiency against nitrophenol reduction [180]. In a similar manner, Kamal et al. reported in situ synthesis of copper nanoparticles (CuNPs) on BCNFs stabilized with carboxy methyl cellulose (CMC), by following a microwave heating approach. The catalytic performance of prepared CMC-Cu-BC was investigated in MB and 4-nitrophenol reduction reactions [181]. Li et al. utilized a laccase modified-oxidized BC membrane as a matrix for TiO2 nanoparticles, which showed excellent efficiency for bio-catalytic and photo-catalytic degradation of textile dye, reactive red X-3B [182]. Patel et al. investigated a rotating catalyst contact reactor (RCCR) for hydrogen gas-assisted de-chlorination of pentachlorophenol, where palletized BC was assimilated on acrylic discs [183]. Jiang et al. studied BC as a scaffold for bismuth oxy bromide (BiOBr) for the photocatalytic degradation of rhodamine B dye. BC nanofibers controlled the particle size of BiOBr by providing a large number of active sites for their attachment, as well as preventing them from agglomeration as a result of confinement to their respected pore [184].

## 6. Conclusions and Future Perspectives

The synthesis processes, commercially used production media, and capital costs for scaled-up production are the main restrictions for BC commercialization. Multiple strategies have attempted to resolve these challenges, the most remarkable of which is the exploration of alternative cheap and recyclable BC production media. A number of alternative production media, such as waste fruits and vegetables, industrial effluents, agricultural wastes, etc., have shown remarkable potential as BC production media and are able to reduce the high scale production costs. Utilization of this waste results in subsequent benefits of reduction in environmental pollution and disposal costs.

Cost reduction directly influences the broad-spectrum applications of BC in multiple fields, including BC being conventionally used as a raw material in some traditional food sources, being additionally utilized as a food additive, stabilizer, fat replacement, and in food packaging applications. Its applications in food industries are extending, owing to its nontoxic fibrous nature. Additionally, its porous structure and gel-shaped appearance advocates its use as a filter in various environmental applications. Due to its application in organic pollutant absorbers, air and water purification, heavy metal absorbents, and catalytic supports for pollutant degradation, the demand for BC has remarkably increased on an industrial scale. Therefore, it is of prime importance to carry out parallel research regarding cost reduction, together with exploring its novel applications.

## Data Availability

Not applicable.
